# Bone Age Determination of Epiphyseal Fusion at Knee Joint and Its Correlation with Chronological Age

**DOI:** 10.3390/medicina60050779

**Published:** 2024-05-08

**Authors:** Jihad A. M. Alzyoud, Eman Rababah, Mohammad H. O. Almuhaisen, Aiman I. Al-Qtaitat

**Affiliations:** 1 Faculty of Medicine, The Hashemite University, Zarqa 13133, Jordan; 2 Department of Basic Dental Sciences, Faculty of Dentistry, The Hashemite University, Zarqa 13133, Jordan; 3 Faculty of Applied Medical Sciences, The Hashemite University, Zarqa 13133, Jordan; eaman@hu.edu.jo; 4 Department of Orthopedic Surgery, Al-Karak Governmental Hospital, Al-Karak 11118, Jordan; mhaisn1@gmail.com; 5 Department of Anatomy and Histology, Faculty of Medicine, Mutah University, Karak 61710, Jordan; aimanaq@mutah.edu.jo; 6 Faculty of Dentistry, Zarqa University, Zarqa 13110, Jordan

**Keywords:** chronological age, X-ray, epiphysis, knee joint, ossification center, bone

## Abstract

*Background and Objectives:* Bone age determination is a valuable method for forensic and disaster identifications of unknown human remains, as well as for medical and surgical procedural purposes. This retrospective research study aimed to determine the age based on epiphyseal fusion stages and investigate differences related to gender. *Materials and Methods:* X-rays of the knee were collected from medical imaging centers in hospitals in the south of Jordan and examined by two observers who determined the bone epiphyseal phase of closure for the femur, tibia, and fibula bone ends close to the knee based on a three-stage classification. *Results:* The main results revealed that females showed earlier epiphyseal union (Stage II) at the lower end of the femur and the upper ends of the tibia and fibula compared to males. In males, the start of complete union (Stage III) at knee bones was seen at the age of 17–18 years, while in females, it was seen at the age of 16–17 years. Additionally, knee bones showed complete union in 100% of males and females in the age groups 21–22 years and 20–21 years, respectively. Although females showed an earlier start and end of epiphyseal complete union than males, analysis of collected data showed no significant age differences between males and females at the three stages of epiphyseal union of the knee bones. *Conclusions:* Findings of the radiographic analysis of bone epiphyseal fusion at the knee joint are a helpful method for chronological age determination. This study supports the gender and ethnicity variation among different geographical locations. Studies with a high sample number would be needed to validate our findings.

## 1. Introduction

Bone growth is a bodily process phenomenon that provides invaluable data about human body physiology and determines its anatomy. Bone growth can be considered a chronological process that follows a schedule that is influenced by several factors related to prenatal, natal, and postnatal phases such as genetic, nutritional, and environmental factors [1]. One important aspect of bone growth is the timing of epiphyseal plate union of long bones, which determine their final length [2]. Bone age determination based on union of epiphyseal growth plate is reliable for forensic, clinical, and biomedical studies [3]. In forensic medicine, several methods have been used to determine the age of a person based on the available bone remains or data extracted from several imaging techniques such as radiography, X-ray, CT scanning, and MRI [4].

Our previous data showed that the epiphysis of the distal ends of female forearm bones closed earlier than in males by two years, as observed in X-ray images [5]. Another study suggested the potential use of clavicular sternal end closure of the epiphyseal plate for age determination in individuals under 30 years of age; however, this was based on a wide range estimation and used 380 images [6]. Bone maturation as depicted by the chronology of epiphyseal union varied based on age, sex, and geography [7]. Bone age determination also varies by the ethnicity and socioeconomic level of the sample [8]. In 2019, an Indian Bengali study reported that knee bone epiphyseal union occurred earlier than the age of 18 years [9]. In another study, a five-stage classification developed and modified previously based on X-ray images was utilized (Schmeling et al., 2004) [8]. This five-stage classification was applied to an MRI image of the knee bones in forensic medicine to determine the age of a person based on their bone features, namely epiphysis fusion, and to match the recommendations of the Group on Forensic Age Diagnostics (AGFAD) (Vieth et al., 2018) [10]. A new study has proven the validity of the AGFAD recommendations for using MRI images in age estimation [11]. Results for the timing of epiphyseal union showed more asymmetry in upper limb bones than in lower limb bones [12].

The knee region forms a complex articulation between the bones of the leg and the thigh. Embryologically, it develops at the age of six weeks, with condensation of cells and ossification centers present by the end of the twelfth week [13]. Bone maturation is determined by changes in the epiphyseal growth plates, which culminate in a line of fusion, and these changes are genetically determined and follow a chronology [14]. These changes can be visualized using different imaging techniques, such as X-ray and MRI. Moreover, data extracted from these images of bone development can be used to predict the age of a bone [14,15]. A recent study using MRI images concluded that bone maturation occurs earlier in females in the proximal tibia than in males or in the distal femur, respectively [16]. Another review of knee age assessment study based on MRI images revealed that methodology is a key point in age estimation, which should be tailored to the staging of union, imaging parameters, exact age, and demographic features of participants [14]. The maturation of the knee is determined by several factors such as genetics, environment, medical conditions, gender, and nutrition [15,17], and an MRI study reported that epiphyseal fusion occurs before the age of 18, with gender differences evident in data analysis [9,18]. It is apparent that different grading systems have been used to describe the events of epiphyseal fusion. The importance of using knee bones in age estimation is overlooked and less compared to other bones such as skull, teeth, and hands bones [LL]. A recent review highlighted the importance of establishing a population- specific criterion for chronological estimates of bone age, which will be valuable in forensic issues, clinical applications, and biomedical studies. Moreover, understanding the effect of geography and ethnicity for each population will deepen our understanding of the different factors that influence the variations evidenced in literature [LL]. In Jordan, research has been investigating the age estimation of different bones, such as hand and skull bones, using different imaging techniques [5]; however, knee bones have not been investigated in the Jordanian population. The main objective of this research was to determine the age of the knee bones based on the fusion of the epiphyses of the distal end of the femur and proximal ends of the tibia and fibula using radiographic images of the knee region.

## 2. Materials and Methods

This was a retrospective cross-sectional study using X-ray images of the knee region and the demographic features of participants. Ethical approval was obtained from the Faculty of Medicine Ethics Committee at Mutah University. X-ray images of the frontal and lateral views of the knee joint were collected from the archives of medical imaging centers and local hospitals in Jordan. Radiographic electronic images of 62 healthy males and 60 healthy females aged 10–22 years, along with their corresponding demographic features including age, gender, and health status, were obtained (Table 1) and all images collected were fully anonymized. Participants included in the study were healthy subjects with no musculoskeletal pathology or trauma, and images with low quality or without date of imaging were excluded. X-ray images were assessed by one experienced physician and a radiologist.

### 2.1. Bone Age Assessment

**Figure 1:**
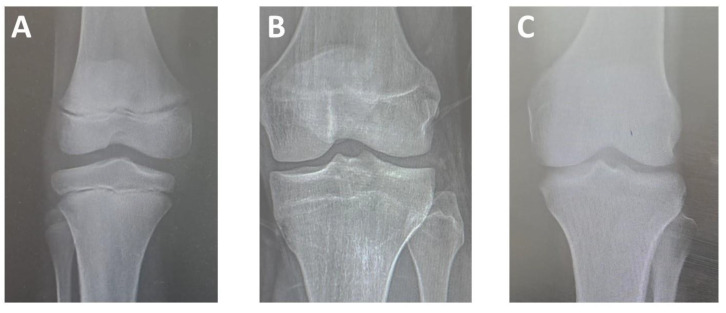
Plain X-ray images of the knee joint showing stages of epiphysis union; (**A**) Stage I, epiphysis is not fused at the distal end of the femur and the proximal ends of the fibula and tibia; (**B**) Stage II: epiphysis is fully ossified, and epiphyseal scar is visible at the distal end of the femur and the proximal end of the tibia; (**C**) Stage III, epiphysis is fully ossified, and epiphyseal scar is not visible at the distal end of the femur and the proximal end of the tibia [12].

Epiphyseal union was estimated based on the three-level criteria proposed by Cameriere et al. (2012) [12], in which the classification is determined by the degree of epiphyseal ossification and the visibility of the epiphyseal scar, divided into three stages (Figure 1). The epiphyses of the femur, tibia, and fibula near the knee joint (i.e., distal end of the femur, proximal end of the tibia, and proximal end of the fibula) were evaluated from all views. Participants’ chronological age calculations were based on the date of the image and the date of birth. The observers were blinded to the age and sex of all radiographs when determining the stage of epiphyseal union for all bones, and for each stage, a score was assigned. Data from all bones (femur, tibia, and fibula) were averaged. In cases of doubt regarding the union stage, the lower stage was adopted.

### 2.2. Statistical Analysis

Statistical analysis was performed using IBM Statistical Package for the Social Sciences software (SPSS, version 20) and Excel Microsoft Office software 2016. Data extracted from images were presented in tables showing the mean age in years for the three stages of union at each of the knee epiphyses for the femur, tibia, and fibula in males and females. Data distribution was tested using the Shapiro–Wilk test. Parametric tests (i.e., ANOVA and the T-test) were employed for normally distributed data while non-parametric tests (i.e., the Kruskal–Willis test) were applied on non-normally distributed data. Levene’s test was used to determine the equality of variances. Statistical significance was determined at a *p*-value limit of 0.05 for sex differences (male vs. female). The results collected were analyzed and compared with similar data collected in other geographical regions worldwide.

## 3. Results

The age and gender distribution of all participants are presented in Table 1. The frequency distribution of males and females at each stage of epiphyseal union with respect to age groups is presented in Table 2 and Table 3, respectively. Mean of age in years for each stage of union at each of the knee epiphyses for femur, tibia, and fibula in males and females are presented in Table 4. For all three epiphyses (the lower end of the femur and the upper ends of both the tibia and fibula), there was an increase in the development of epiphyseal closure through all stages of union with increasing chronological age. The one-year life span distribution was wide enough within each age group to show varied changes in epiphyseal union. It should be noted that for each age group included in our study, for example, 14–15 years, it indicates the completion of a certain number of years (14 years) and not the completion of the next number of years (15 years).

### 3.1. Lower End of Femur

Table 2 and Table 3 display the distribution of male subjects across 10 age groups at each stage of union. No epiphyseal union was observed in males up to the age of 15–16, while the onset of complete union was noted in the age group 17–18. In females, the onset of complete union occurred earlier in the age group 16–17. All subjects exhibited complete union at the lower end of the femur in the age groups 21–22 for males and 20–21 for females, respectively. Regarding both males and females, the distal femur showed complete epiphyseal union (stage III) in a total of 50 out of 122 cases examined (41.0%), distributed between the ages of 17 and 22 in males and 16 and 22 in females. Despite females showing an earlier onset of epiphyseal complete union compared to males, analysis of the collected data revealed no significant age differences between males and females at the three stages of epiphyseal union at the distal end of the femur (T-test, *p* value > 0.05) (Table 4) (Supplementary Materials Table S1).

### 3.2. Upper End of Tibia and Fibula

Data presented in Table 2 and Table 3 reveal that there was no epiphyseal union for either the tibia or fibula in age groups 12–15 for males and 12–14 for females. The youngest age groups showing complete union of the tibia and fibula in males and females were 17–18 and 16–17, respectively. Complete union (stage III) for both the tibia and fibula in males was observed in all cases at 21–22 years, while in females, complete union for both bones was observed in the age group 20–22 (Table 2 and Table 3). Although females showed an earlier onset of epiphyseal complete union than males, analysis of the data collected showed no significant age differences between males and females at the three stages of epiphyseal union of the upper ends of both the tibia and fibula (T-test, *p* value > 0.05) (Table 4) (Supplementary Materials Table S1).

## 4. Discussion

Main results revealed that females showed an earlier onset of epiphyseal complete union (Stage III) compared to males. Similarly, males exhibited an earlier onset of epiphyseal fusion at the age of 15 years at the lower end of the femur compared to the upper ends of the tibia and fibula, which begin at the age of 16 years, whereas in females, all knee bones began epiphyseal fusion at the age of 15 years. However, this gender difference was insignificant at the three stages of epiphyseal union of the knee bones. In males, the start of complete union (Stage III) at knee bones was seen at the age of 17–18 years, while in females, it was seen at the age of 16–17 years. Additionally, knee bones showed complete union in 100% of males and females in the age groups 21–22 years and 20–21 years, respectively.

Retrospective studies utilizing images generated by different imaging techniques are important tools for bone age determination, providing a large sample size and minimal radiation exposure [19]. Furthermore, geographic differences in epiphyseal fusion among different ethnicities necessitate studies in different geographical areas to aid in forensic, legal, and scientific disciplines [20]. A strength of this study was the use of images from a similar ethnicity and socioeconomic level [9]. Gender differences in epiphyseal fusion reported in previous studies, where females experience early fusion of bone epiphyses, support the findings of this study [9]. All images were captured at the same imaging center at Al KarK hospital. The knee joint offers the advantage of being a large, easily accessible joint with three epiphyses, and it is relatively easy to capture images with minimal artifacts. The knee joint is frequently studied for estimating age and gender [20,21,22]. In this study, the results showed that female images displayed an earlier onset of fusion stage of the proximal tibial and fibular epiphyses compared to male images. Various studies have reported the complete fusion of the distal epiphysis of the femur occurs between the ages of 14 and 18 in males and 16 and 19 in females [20,21,22]. This is consistent with our study, which showed a range of 17–20 years in males and 16–19 years in females.

A recent review of knee age assessment studies based on MRI images revealed that methodology is a key point in age estimation, which should be tailored to include factors such as staging of union, imaging parameters, exact age, and demographic features of participants [14]. An earlier Irish study in 2008 applied five stages to describe the union [23]. In this study, a summary table of literature from different populations showed a similar age range for complete union (Stage III) results that support our findings, while other data showed lower ages than our results. This difference could be explained by the different populations of the corresponding studies [23]. Additionally, our study used three stages for evaluating the development of the knee (epiphyseal union) based on chronological age, while the Irish study applied a five-stage method. A recent study using MRI images concluded that maturation is earlier in females and the proximal tibia than in males or the distal femur, respectively [16]. Knee maturation is determined by several factors such as genetics, environment, medical conditions, gender, and nutrition [15,19]. The study results were similar to those of a previous MRI study, which reported that fusion of the epiphysis occurred before the age of 18 and gender differences were evident in the data analysis [18]. The data findings of this study related to the fibula support the earlier observation that the fibula has a different rate of epiphyseal union compared to the femur and tibia in some populations [23,24].

The importance of using knee bones in age estimation is overlooked and less researched compared to other bones such as skull, teeth, and hands bones [25]. A recent review highlighted the importance of establishing a population-specific criterion for chronological estimates of bone age [25]. This research is the first to be conducted in Jordan for estimation of knee bones and it encountered a number of inherited issues that limit the generalizability of study conclusions and the understanding of variations in epiphyseal fusion. First, the current study design was retrospective, relying on plain X-rays and investigating the impact of gender and age on bone age maturation in the Jordanian population, while other factors were missing due to retrospective data collection, such as nutrition, physical activity, environmental, and underlying health conditions that could influence bone maturation and consequently affect understanding of variations in epiphyseal fusion. Another factor which limits the generalizability of results is the small sample size, which led to biases, and this was attributed to the limited archived resources at the age range. It is highly recommended that future studies with a prospective design and with large sample size are carried out to minimize inherited biases and allow more precise tracing of the progression of epiphyseal fusion in individuals [26]. Furthermore, radiographic findings of this study would provide a basis for the use of other techniques such as MRI and ultrasound images, and allow the comparison and exploration of more subjects.

Moreover, the literature revealed that several grading systems for epiphyseal fusion were utilized and targeted samples were confined to certain geographic locations within a country, while other studies were applied to whole population in a country. In addition, there was no consensus on unified staging and method design, although different imaging methods were used for living subjects and skeletal remains [14]. In this study, a three-stage classification of the epiphyseal union was used, instead of the more common five-stage classification employed in previous studies [23,27]. Although a five-stage method clearly defined the process as a dynamic continuous process of epiphyseal closure, a three-stage method clearly define the process into three phases, a non-fusing phase, a fusing phase, and a complete phase with emphasis on the complete union definition, and this would minimize the overlapping between phases, which makes the statistical analysis more consistent [22,23,27]). On the other hand, developing a classification scale that takes into consideration the different factors influencing the stages of epiphyseal fusion could provide more precise insights into bone maturation [28].

Recently, a three-point level of epiphyseal union was utilized to estimate the age of knee bones using ultrasound. The study concluded it was a reliable classifying method for bone age estimation and its results were comparable to those of MRI image estimates [28]. Another recent study evaluated the effect of using two types of imaging methods (i.e., X-rays and MRI) by applying a four-stage epiphyseal union and concluded that both methods yielded similar outcomes of age estimations. However, it found MRI showed better prediction of models and was safer than X-ray [29]. A recent review highlighted the impact of heterogeneity in studies using knee MRI images as a predictive method for determining bone age. It found there was potential for false predictions, especially among young people [14]; however, this could be improved by introducing computed aided measurements and AI [26].

The current study focused on subjects from the Jordanian population of different areas with the aim of providing preliminary data on bone age determination to help in forensic and disaster identifications of unknown human remains, as well as being potentially applicability for medical and surgical procedural purposes. Several studies have found the differences between ethnicities may limit the applicability of the results to other populations due to genetic, nutritional, and environmental differences. However, they provide an opportunity to compare findings with those of studies on other populations, which will lead to an exploration of global differences between different populations in epiphyseal fusion and enhance the applicability of findings in forensic and clinical contexts as well as deepen our understanding of the variations reported.

## 5. Conclusions

The current study presents valuable information about the age of knee epiphysis union and potential differences between males and females among young Jordanian individuals based on knee X-rays in anteroposterior and lateral orientations. These data could serve as a foundation for modeling artificial intelligence systems, offering potential for future research. Additionally, integrating these findings with data from other bones in various contexts could enhance the accuracy of age estimation. Moreover, conducting similar studies on diverse ethnicities to explore epiphyseal fusion among populations would contribute significantly to forensic, legal, and scientific fields.

## Figures and Tables

**Table 1:** Age and sex distribution of all subjects.

Age (Years)	Males	Females	All
12–13	2	3	5
13–14	3	3	6
14–15	5	4	9
15–16	7	9	16
16–17	7	8	15
17–18	8	6	14
18–19	7	6	13
19–20	7	6	13
20–21	7	7	14
21–22	9	8	17
Total	62	60	122

**Table 2:** Male subjects’ distribution at each stage of epiphyseal union in the lower ends of femur, upper ends of tibia and fibula for each age group; I = non-union, II = non-full union, III = complete union.

Age (Years)	Number of Participants	Femur Stage			Tibia Stage			Fibula Stage			Total
		I	II	III	I	II	III	I	II	III	
12–13	2	2	-	-	2	-	-	2	-	-	6
13–14	3	3	-	-	3	-		3	-	-	9
14–15	5	5	-	-	5	-	-	5	-	-	15
15–16	7	6	1	-	7	-	-	7	-	-	21
16–17	7	4	3	-	6	1	-	5	2	-	21
17–18	8	2	3	3	3	3	2	2	3	3	24
18–19	7	2	1	4	1	4	2	2	3	2	21
19–20	7	-	2	5	-	3	4	-	2	5	21
20–21	7	-	2	5	-	1	6	-	2	5	21
21–22	9	-	-	9	-	-	9	-	-	9	27
Total	62	24	12	26	27	12	23	26	12	24	186

**Table 3:** Female subjects’ distribution at each stage of epiphyseal union in the lower ends of femur, upper end of tibia and fibula for each age group; I = non-union, II = non-full union, III = complete union.

Age (Years)	Number of Participants	Femur Stage			Tibia Stage			Fibula Stage			Total
		I	II	III	I	II	III	I	II	III	
12–13	3	3	-	-	3	-	-	3	-	-	9
13–14	3	3	-	-	3	-		3	-	-	9
14–15	4	4	-	-	4	-	-	4	-	-	12
15–16	9	7	2	-	6	3	-	7	2	-	27
16–17	8	3	4	1	6	1	1	5	2	1	24
17–18	6	2	3	1	1	3	2	1	3	2	18
18–19	6	-	3	3	-	4	2	-	5	1	18
19–20	6	-	2	4	-	3	3	-	3	3	18
20–21	7	-	-	7	-	-	7	-	-	7	21
21–22	8	-	-	8	-	-	8	-	-	8	24
Total	60	22	14	24	23	14	23	23	15	22	180

**Table 4:** Mean of age (years) for each stage of union at each of the knee epiphyses for femur, tibia, and fibula in males and females.

Bone	Union Stage	Gender	Number	Mean Age (Years) ± Std. Deviation
Femur Epiphyseal Fusion Stage	Stage I	Male	24	14.88 ± 1.676
		Female	22	14.45 ± 1.503
	Stage II	Male	12	17.50 ± 1.679
		Female	14	16.93 ± 1.328
	Stage III	Male	26	19.50 ± 1.421
		Female	24	19.63 ± 1.408
Tibia Epiphyseal Fusion Stage	Stage I	Male	26	14.93 ± 1.542
		Female	23	14.52 ± 1.473
	Stage II	Male	12	18.00 ±1.128
		Female	15	17.21 ± 1.477
	Stage III	Male	24	19.78 ±1.313
		Female	22	19.61 ± 1.500
Fibula Epiphyseal Fusion Stage	Stage I	Male	26	14.92 ± 1.623
		Female	23	14.48 ± 1.442
	Stage II	Male	12	17.92 ± 1.379
		Female	15	17.33 ± 1.345
	Stage III	Male	24	19.63 ± 1.408
		Female	22	19.68 ± 1.492

## Data Availability

The raw data of this article will be made available by the corresponding author on request.

## Supplementary Materials

The following supporting information can be downloaded at: https://www.mdpi.com/article/10.3390/medicina60050779/s1, Table S1: Statistical analysis of gender differences between the different stages of femur epiphyseal fusion; Table S2: Statistical analysis of gender differences between the different stages of tibia epiphyseal fusion; Table S3: Statistical analysis of gender differences between the different stages of fibula epiphyseal fusion.
