# Supplementary material for: Bone Age Determination of Epiphyseal Fusion at Knee Joint and Its Correlation with Chronological Age
Source: Medicina (Kaunas). 2024 May 8;60(5):779. doi: 10.3390/medicina60050779 (PMC11122822; doi:10.3390/medicina60050779)
Supplement: Supplementary file 1 [file medicina-60-00779-s001.zip › medicina-2958880-supplementary.pdf]

Table S1: Statistical analysis of gender differences between the different stages of Femur Epiphyseal Fusion

Independent Samples Test												
Femur Epiphyseal Fusion Stage			Levene's Test for Equality of Variances		t-test for Equality of Means							
			F	Sig.	t	df	Significance		Mean Difference	Std. Error Difference	95% Confidence Interval of the Difference	
							One-Sided p	Two-Sided p			Lower	Upper
Stage I	Age	Equal variances assumed	.089	.767	.893	44	.188	.377	.420	.471	-.529	1.370
		Equal variances not assumed			.897	43.983	.187	.375	.420	.469	-.524	1.365
Stage II	Age	Equal variances assumed	1.293	.267	.969	24	.171	.342	.571	.590	-.646	1.789
		Equal variances not assumed			.951	20.882	.176	.352	.571	.601	-.678	1.821
Stage III	Age	Equal variances assumed	.211	.648	-.312	48	.378	.756	-.125	.401	-.930	.680
		Equal variances not assumed			-.312	47.748	.378	.756	-.125	.400	-.930	.680

Table S2: Statistical analysis of gender differences between the different stages of Tibia Epiphyseal Fusion

Independent Samples Test												
Tibia Epiphyseal Fusion Stage			Levene's Test for Equality of Variances		t-test for Equality of Means							
			F	Sig.	t	df	Significance		Mean Difference	Std. Error Difference	95% Confidence Interval of the Difference	
							One-Sided p	Two-Sided p			Lower	Upper
Stage I	Age	Equal variances assumed	.017	.896	.943	48	.175	.351	.404	.429	-.458	1.266
		Equal variances not assumed			.946	47.342	.174	.349	.404	.427	-.455	1.263
Stage II	Age	Equal variances assumed	1.682	.207	1.503	24	.073	.146	.786	.523	-.293	1.864
		Equal variances not assumed			1.535	23.728	.069	.138	.786	.512	-.271	1.843
Stage III	Age	Equal variances assumed	.356	.554	.418	44	.339	.678	.174	.416	-.664	1.011
		Equal variances not assumed			.418	43.243	.339	.678	.174	.416	-.664	1.012

Table S3: Statistical analysis of gender differences between the different stages of Fibula Epiphyseal Fusion

Independent Samples Test												
Fibula Epiphyseal Fusion Stage			Levene's Test for Equality of Variances		t-test for Equality of Means							
			F	Sig.	t	df	Significance		Mean Difference	Std. Error Difference	95% Confidence Interval of the Difference	
							One-Sided p	Two-Sided p			Lower	Upper
Stage I	Age	Equal variances assumed	.038	.847	1.009	47	.159	.318	.445	.441	-.442	1.332
		Equal variances not assumed			1.016	46.998	.157	.315	.445	.438	-.436	1.326
Stage II	Age	Equal variances assumed	.002	.961	1.107	25	.139	.279	.583	.527	-.502	1.668
		Equal variances not assumed			1.104	23.447	.140	.281	.583	.528	-.508	1.675
Stage III	Age	Equal variances assumed	.012	.913	-.133	44	.447	.895	-.057	.428	-.919	.805
		Equal variances not assumed			-.132	43.073	.448	.895	-.057	.429	-.922	.808
